# On Some Anomalous Cases of Locomotor Ataxy

**Published:** 1905-09

**Authors:** F. H. Edgeworth

**Affiliations:** Professor of Medicine at University College, Bristol; Assistant-Physician to the Bristol Royal Infirmary


### ON SOME ANOMALOUS CASES OF LOCOMOTOR ATAXY.

BY

F. H. Edgeworth, M.B. Cantab, D.Sc. Lond.,

*Professor of Medicine at University College, Bristol; Assistant-Physician to the Bristol Royal Infirmary.*

---

Among the large number of cases of locomotor ataxy conforming to the generalised description of the disease as given in text-books occur some which deviate from this in some way or other—either by the absence of ordinary features or by the presence of rare ones, or by the co-existence of other diseases which modify the usual clinical picture of the disease.

Absence of the knee-jerks is an almost constant feature of the malady. They may, however, be present either because the lumbar region of the spinal cord is not affected, or owing to intercurrent degeneration of the lateral pyramidal tracts. In the former class of cases a diagnosis may be made if other features, especially lightning pains and gastric crises, are present which are almost, if not quite, limited to this disease.

For instance, a man, aged 41, who eight years previously had primary and secondary syphilis, said that for a year he had suffered from intense shooting pains in the muscles of the legs, occurring in attacks lasting from four hours to several days. His description of these pains was so characteristic that a diagnosis of tabes could be made, though the physical signs were absolutely negative. He was ordered five grains of aluminium chloride three times a day, with antifebrin during the attacks. Six months later he reported that the pains, though still occurring, were less intense, and that the attacks were not so frequent or prolonged. The physical signs were still negative.

Gastric and intestinal crises, too, may be so definite and characteristic that a diagnosis of tabes may be made even in the absence of physical signs. Thus a man, aged 42, who had had a hard chancre, though no secondary or tertiary symptoms—owing to persistent treatment with mercury—thirteen years previously, began to suffer six years afterwards from lightning pains, and three years after that from gastric crises. These attacks lasted from two days to as long as ten weeks, with variable intervals between them. They began quite suddenly, and consisted of loss of appetite and frequent vomiting, first of blood and then of clear fluid. The vomiting might take place as frequently as twelve times in the twenty-four hours, and during that time two quarts of fluid might be brought up. Each attack was attended with intense pains in the epigastrium, but without the slightest tenderness on pressure. The temperature during the attacks did not rise above 99° F. Immediately an attack was over the appetite became exceedingly good, and the weight lost was rapidly regained. I saw him during an attack. The knee-jerks were present and equal, the plantar reflexes present and flexor in type; the pupils, rather large, reacted both to light and accommodation, and there was no visual defect or contraction of the fields of vision; Romberg's symptom was absent; there was no zone of skin anæsthesia or tenderness of the abdomen.

The attacks of vomiting in this case were typical of those of gastric crises, in particular the association of repeated vomiting and intense pain in the stomach without any tenderness on pressure, and the diagnosis was supported by the presence of lightning pains. The only drug which controlled the attacks was morphia administered hypodermically. Three years subsequently the gastric crises gradually disappeared, though the lightning pains continued, and, as so often happens in these cases, the man had developed a tendency to become a morphino-maniac. The only physical evidence of tabes which had developed was Romberg's sign.

The case also illustrated the fact that some general systematic syphilitic infection took place, though the only outward sign of the disease was the initial sore.

Another case of gastric crises was interesting in presenting a combination of symptoms—ocular paralysis, gastric crises, and loss of knee-jerks. It was that of a married woman, aged 27, who five years previously had suffered from symptoms very suggestive of syphilis. For two years she had had lightning pains in the legs, and ten or eleven attacks of repeated vomiting accompanied by severe pain in the stomach. For one week she had also suffered from double vision when both eyes were open. On examination it was found that the knee-jerks were absent and that the left external rectus muscle was partially paralysed; the pupils reacted to light and accommodation, and there were no other physical signs of tabes. Under the administration of iodide the ocular paralysis cleared up in two months, but the other signs and symptoms persisted. She was seen on several occasions during an attack of vomiting, and the absence of any superficial or deep tenderness of the abdomen confirmed.

I have not met with any case of intestinal crises with present knee-jerks, but in the following case they were the first symptom of the disease. A man, aged 44, who had had a hard chancre twenty years previously, suffered from severe intermittent pain in the abdomen, each attack lasting from two to ten hours, two or three times a week. During the attacks there was no vomiting and no tenderness on pressure on the abdomen, but the bowels were never opened during their occurrence. These attacks, when I first saw him, had lasted about a twelvemonth, and had obliged him to give up work. For nine months he had also had lightning pains in the legs, and for seven slight incontinence of urine. On examination it was found that the knee-jerks were absent, Romberg's sign present, whilst the pupils, of moderate size, reacted to light and accommodation. He was examined on several occasions during an attack of pain, when the abdomen was found to be of natural shape, and there was no tenderness on pressure. He was given ♏ v. of tinct. cannab. indic., ♏ iii. of liq. arsen., and gr. ii. of alum. chlorid. three times a day after meals. This fortunately gave relief; the attacks gradually became less acute and shorter in duration, so that in six months' time he was able to resume work.

Transient paralysis of one or more of the external ocular muscles may, as is well known, be an early symptom of a case of tabes. In some cases, as in the following, it may be the first symptom—occurring before even the loss of knee-jerks. A man, aged 35, who had had syphilis eleven years before, complained of drooping of the left upper eyelid. On examination there was ptosis, but no paralysis of the left superior rectus or other muscle of the eye. No sign or symptom of tabes was present. The ptosis disappeared under the influence of mercury inunctions. Three years later he had paresis of the left rectus externus. It was then found that the knee-jerks could only be obtained on reinforcement.

The knee-jerks if lost in tabes may return in consequence of an attack of hemiplegia. This was first pointed out by Hughlings Jackson and Taylor, who published a case[1] in which this occurred, and in which four years later the knee-jerk on the non-paralysed side had again disappeared, whilst that on the paralysed side had become very much less, and they prophesied that later on the knee-jerk on the paralysed side would also disappear. Such a case in which the knee-jerks had both again been lost I published[2] a little later. The cause of the reappearance of the jerks is the reinforcement brought about by the pyramidal degeneration consequent on the cerebral lesion, whilst their subsequent disappearance is due to the progressive atrophy of the posterior root fibres.

The following case is of interest in regard to this matter. A man, aged 63, had suffered from syphilis thirty years before, and for seventeen years had pains in the legs, in attacks of variable duration, as if the muscles were pinched in a vice. Four years before coming under observation he had an attack of left hemiplegia, affecting the face, arm and leg. On examination he was found to have smallish pupils, reacting to accommodation but not to light. Romberg's symptom was absent. There was slight weakness in the lower part of the left side of the face, but practically none in the left arm or leg. The left triceps jerk was present, the right absent. Both knee-

[1] *Brit. M. J.*, 1891, ii, 57; 1894, i, 1350.

[2] *Ibid.*, 1899, ii, 853.

jerks were present, the right one being less marked than the left. There was no knee or ankle clonus. The presence of tabes was shown by the lightning pains and Argyll-Robertson pupils occurring in a man who had had syphilis, the presence of past hemiplegia by the paresis of the left side of the face and by the tendon-jerks above mentioned. The pyramidal degeneration was thus in evidence, though the motor symptoms had almost disappeared.

In connection with this possibility of the reappearance of knee-jerks lost owing to tabes, it is interesting to note that an arthritis does not bring them back. An arthritis, as is well known, produces a reflex atrophy of the extensor muscles passing over the joint and an increase of their myotatic irritability if the spinal cord is intact. But if an arthritis of the knee supervene in a case of tabes with lost knee-jerks, whether it be a tabetic arthropathy or due to other causes, the knee-jerk does not reappear on that side.

The following case is an illustration of what at first sight seemed to be an exception to this rule. A woman, aged 58, who twenty-four years previously had had symptoms very suggestive of syphilis, complained of stiffness of the left shoulder of six months' duration. She was found to have Argyll-Robertson pupils of medium size, absent knee-jerks, and static ataxy especially marked when the eyes were shut. There was no inco-ordination or defect of cutaneous or muscular sensibility in the arms. The left shoulder joint was enlarged and tender, with considerable limitation of movement and partial atrophy of the muscles passing over the joint. The left biceps-jerk was very brisk, the triceps-jerk absent. No tendon jerks were present in the right arm. On skiagraphic examination the head of the left humerus was seen to be distinctly enlarged though of normal shape and of the same density as on the right side. The arthritis—apparently of a rheumatoid nature—had reinforced the biceps owing to the fact that there was no tabetic affection of the upper part of the spinal cord.

Peripheral neuritis may complicate a case of tabes, as in the following case. A man, aged 34, who had had syphilis ten years before, suffered for the five years before coming under notice from sharp, shooting pains in the arms and legs, which he attributed to rheumatism, as they were always worse in wet weather. For four years he had also suffered from ataxy, so that he could not go out in the evening and had to perform his ablutions sitting down. A year before coming under treatment his right foot "dropped," he could not dorsiflex it, and was very liable to trip up whilst walking; this gradually improved and got well in six months. A fortnight previously his right wrist "dropped," and he was unable to use his hand. On examination it was found that the knee-jerks and plantar reflexes were absent, cutaneous sensibility was deficient in the lower part of the legs and feet, Romberg's symptom was very marked, and that the pupils reacted to accommodation but not to light. In addition, there was paralysis of the extensors of the right wrist, thumb, and fingers, with loss of tone and electrical reactions of degeneration. There was partial anæsthesia of the outer and posterior surfaces of the forearm and back of the hand, thumb, and fingers. The nerves were not tender on pressure.

The patient was treated with large doses of iodide, massage, and electricity, and in two months almost complete recovery took place. The physical signs of tabes did not change.

The nature of this intercurrent peripheral neuritis, affecting first the leg and then the arm, was not apparent—possibly it was of syphilitic origin.

